# CUMYL-4CN-BINACA Is an Efficacious and Potent Pro-Convulsant Synthetic Cannabinoid Receptor Agonist

**DOI:** 10.3389/fphar.2019.00595

**Published:** 2019-05-29

**Authors:** Richard C. Kevin, Lyndsey Anderson, Iain S. McGregor, Rochelle Boyd, Jamie J. Manning, Michelle Glass, Mark Connor, Samuel D. Banister

**Affiliations:** ^1^School of Psychology, Faculty of Science, The University of Sydney, Sydney, NSW, Australia; ^2^Brain and Mind Centre, The University of Sydney, Sydney, NSW, Australia; ^3^Lambert Initiative for Cannabinoid Therapeutics, The University of Sydney, NSW, Australia; ^4^Discipline of Pharmacology, Faculty of Medicine and Health, The University of Sydney, Sydney, NSW, Australia; ^5^Faculty of Medicine and Health Sciences, Macquarie University, Sydney, NSW, Australia; ^6^Department of Pharmacology and Toxicology, The University of Otago, Dunedin, New Zealand; ^7^School of Chemistry, Faculty of Science, The University of Sydney, Sydney, NSW, Australia

**Keywords:** novel psychoactive substance, new psychoactive substance, CUMYL, synthetic cannabinoid, seizure, convulsant

## Abstract

Synthetic cannabinoid receptor agonists (SCRAs) are the largest class of new psychoactive substances (NPS). New examples are detected constantly, and some are associated with a series of adverse effects, including seizures. CUMYL-4CN-BINACA (1-(4-cyanobutyl)-*N*-(2-phenylpropan-2-yl)indazole-3-carboxamide) is structurally related to potent, cumylamine-derived SCRAs such as 5F-CUMYL-PINACA, but is unusual due to a terminal aliphatic nitrile group not frequently encountered in SCRAs or pharmaceuticals. We report here that CUMYL-4CN-BINACA is a potent CB_1_ receptor agonist (*K*_i_ = 2.6 nM; EC_50_ = 0.58 nM) that produces pro-convulsant effects in mice at a lower dose than reported for any SCRA to date (0.3 mg/kg, i.p). Hypothermic and pro-convulsant effects in mice could be reduced or blocked, respectively, by pretreatment with CB_1_ receptor antagonist SR141716, pointing to at least partial involvement of CB_1_ receptors *in vivo*. Pretreatment with CB2 receptor antagonist AM-630 had no effect on pro-convulsant activity. The pro-convulsant properties and potency of CUMYL-4CN-BINACA may underpin the toxicity associated with this compound in humans.

## Introduction

CUMYL-4CN-BINACA (1-(4-cyanobutyl)-*N*-(2-phenylpropan-2-yl)indazole-3-carboxamide) (**1**), also known as 4CN-CUMYL-BUTINACA, CUMYL-CYBINACA, and SGT-78, has been available as a synthetic cannabinoid receptor agonist (SCRA) and new psychoactive substance (NPS) in the European Union and elsewhere since late 2015, with more than 2,400 seizures reported by nine countries (European Monitoring Centre for Drugs and Drug Addiction, 2017; Arıkan Ölmez et al., 2018). CUMYL-4CN-BINACA has been detected in multiple forms, including liquids and powders, with a single seizure of 50 kg of powder intercepted by Spanish customs en route from China (European Monitoring Centre for Drugs and Drug Addiction, 2017). Eleven fatalities with analytical confirmation of CUMYL-4CN-BINACA have occurred in the EU, with CUMYL-4CN-BINACA attributed as cause or contributor to death in at least five of these (EMCDDA, 2017). In Turkey, CUMYL-4CN-BINACA was detected in 85 post-mortem blood samples collected from autopsies conducted in the latter half of 2016 (Yeter, 2017).

**Figure 1:**
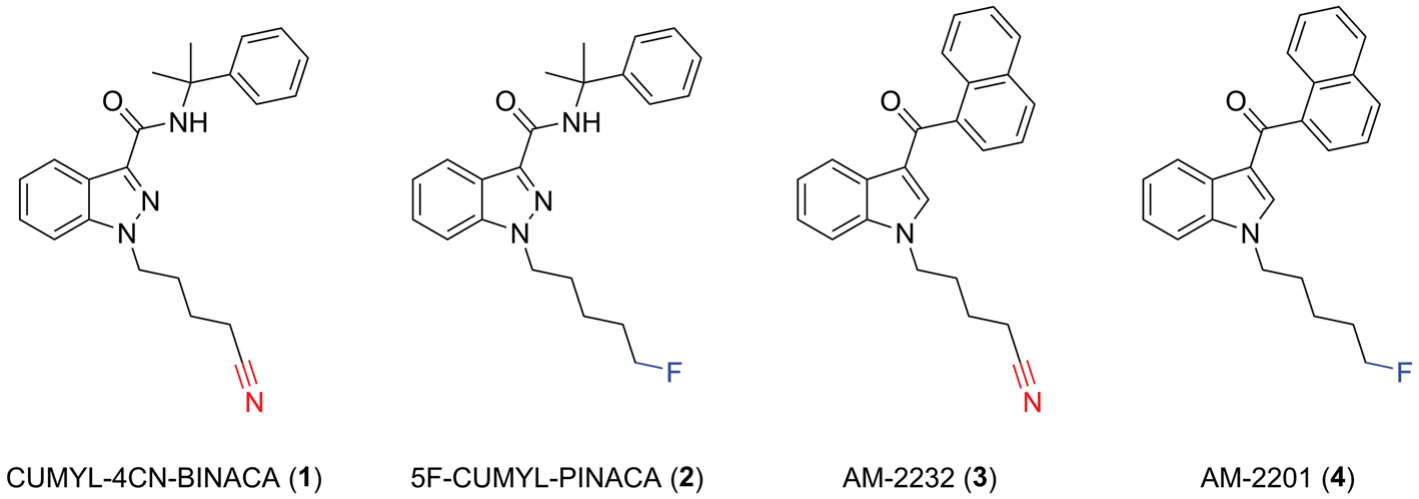
Molecular structures of CUMYL-4CN-BINACA (**1**), CUMYL-5F-PINACA (**2**), AM-2232 (**3**), and AM-2201 (**4**). Note that CUMYL-4CN-BINACA and AM-2232 are the nitrile analogues of CUMYL-5F-PINACA and AM-2201, respectively.

CUMYL-4CN-BINACA originates as example SGT-78 in a New Zealand patent from 2014 describing the preparation of cannabinoids for “treating pain and nausea, stimulating appetite, and inducing a positive mood change,” and its synthetic route is analogous to those reported for related compounds (Bowden and Williamson, 2014; Longworth et al., 2017). The SCRAs CUMYL-PINACA and 5F-CUMYL-PINACA (**2**), containing a pentyl or 5-fluoropentyl chain, respectively, in place of the 4-cyanobutyl group of CUMYL-4CN-BINACA, were reported in several cases of acute intoxication (**Figure 1**; Abouchedid et al., 2017; Dobaja et al., 2017). Unlike CUMYL-4CN-BINACA, no fatalities have been attributed to CUMYL-PINACA or 5F-CUMYL-PINACA. The structural characterization of CUMYL-4CN-BINACA was recently reported (Bovens et al., 2017), and CUMYL-4CN-BINACA metabolites have been identified in authentic urine samples and human liver microsomes (Öztürk et al., 2018; Staeheli et al., 2018).

A nitrile group occurs in more than 30 approved pharmaceuticals; commonly as an aromatic nitrile, but less commonly as an aliphatic nitrile since the latter may release cyanide *in vivo* (Tanii and Hashimoto, 1984). Indeed, CUMYL-4CN-BINACA undergoes apparent metabolic nitrile hydrolysis, which may contribute to toxicity including renal failure (Åstrand et al., 2018; El Zahran et al., 2019). AM-2232 (**3**) is an analogue of the historically prevalent SCRA AM-2201 (**4**) described in a patent granted to Makriyannis and Deng (2001), and featuring a terminal nitrile group, that was previously detected in the German NPS market (Langer et al., 2014). The relatively greater CB_1_ binding affinity of AM-2232 (*K*_i_ = 0.28 nM) compared to AM-2201 (*K*_i_ = 1.0 nM) represents a possible motivation for the introduction of this motif in the case of CUMYL-4CN-BINACA (Makriyannis and Deng, 2001).

The cannabimimetic activity of several cumylamine-derived SCRAs has been described; they are potent CB_1_ receptor agonists *in vitro* and produce robust dose-dependent hypothermia in rodents (Asada et al., 2017; Longworth et al., 2017). Given multiple case reports of acute toxicity associated with CUMYL-4CN-BINACA, we sought to determine the hitherto uncharacterized pharmacological profile of this compound for direct comparison to its prevalent 5-fluoropentyl analogue. In the present study, we describe the binding affinity and functional activity of CUMYL-4CN-BINACA at human CB_1_ and CB_2_ receptors *in vitro*, as well as the cannabimimetic and pro-convulsant properties of this SCRA in mice.

## Materials and Methods

### Chemicals and Reagents

CUMYL-4CN-BINACA was obtained from Cayman Chemical (MI, USA), and SR141716 from Sigma-Aldrich (NSW, Australia). Dulbecco’s modified Eagle’s medium (DMEM) was purchased from Thermo Fisher (MA, USA). Fetal bovine serum (FBS) was obtained from Moregate Biotech (NZ). Cell culture plasticware was sourced from Corning (NY, USA). [^3^H]-CP 55,940, harvest plates, and Irgasafe plus, were all purchased from PerkinElmer (MA, USA). Polypropylene v-bottom mixing 96-well plates were acquired from Hangzhou Gene Era Biotech Co Ltd (China). Branched polyethyleneimine was purchased from Sigma (MO, USA). The Bradford Protein Assay Kit was purchased from BIORAD (CA, USA).

### *In Vitro* Radioligand Binding Assay

Cell membranes expressing the human CB_1_ (hCB_1_) or CB_2_ (hCB_2_) receptor were purified as described previously (Finlay et al., 2017). Briefly, HEK293 cells were stably transfected with human CB_1_ N-terminally tagged with bovine pre-prolactin signal sequence and 3-haemagglutinnin residues as described in Finlay et al. (2017). Similar HEK293 cells expressing a 3-HA tagged CB_2_ receptor were used for CB_2_-rich membrane isolation. Cells were cultured in DMEM + 10% FBS under Zeocin-resistant (250 μg/ml) antibiotic selection. Cells were expanded into approx. 24 × 175 cm^2^ vented-cap plastic culture flasks, and dislodged at maximum confluency using ice-cold 5 mM EDTA. Cells were sedimented and snap frozen at −80°C. The pellet was resuspended in ice-cold sucrose buffer (200 mM sucrose, 50 mM Tris–HCl pH 7.4, 5 mM MgCl_2_, 2.5 mM EDTA) and manually homogenized using a glass pestle and dounce homogenizer. The homogenate was centrifuged at 1,000 × *g* for 10 min. The membrane-rich supernatant was retained and re-centrifuged at 26,916 × *g* for 30 min. The membrane pellet was resuspended in sucrose buffer and protein levels quantified using a Bradford Protein Assay Kit and stored at –80°C.

Competition binding assays were conducted on purified membrane preparations as described previously (Finlay et al., 2017). Concentration dilution series of non-tritiated drugs were prepared in binding buffer (50 mM HEPES pH 7.4, 1 mM MgCl_2_, 1mM CaCl_2_, 2 mg/mL BSA). [^3^H]-CP 55,940 was also diluted to a final concentration of 1 nM (CB_1_) or 2 nM (CB_2_) in binding buffer. Membranes were diluted to 5 μg per assay point. Reagents were mixed at a final assay volume of 200 μl in v-bottom polypropylene 96-well plates and incubated for 1 h at 30°C. Simultaneously, 1.2-μm pore fiberglass filters of a 96-well harvest plate were blocked with a solution of 0.1% w/v branched polyethylenimine. Following incubation, the harvest plate was applied to a Pall vacuum manifold (NY, USA) at 5 mmHg. Wells were washed with 200-μl ice cold wash buffer (50 mM HEPES pH 7.4, 500 mM NaCl, 1 mg/ml BSA). Drug/membrane mixtures were transferred from the v-bottom mixing plate and applied to the harvest plate. Wells of the v-bottom plate were washed with 200-μl ice-cold wash buffer, and the wash was also applied to the respective wells on the harvest plate. Finally, each well on the harvest plate was washed three times with 200 μl of ice-cold wash buffer, then allowed to dry overnight. The underside of the harvest plate was sealed, and 50 μl Irgasafe Plus was applied to each well and read in a Wallac MicroBeta® TriLux liquid scintillation counter (Perkin Elmer, MA, USA) for 2 min per well.

### *In Vitro* Cannabinoid Receptor Functional Assay

Mouse AtT20FlpIn neuroblastoma cells stably transfected with human CB_1_ or CB_2_ have been previously described (Banister et al., 2016) and were cultured in DMEM containing 10% FBS, 100 U penicillin/streptomycin, and 80 µg/ml of hygromycin. Cells were passaged at 80% confluency, cells for assays were grown in 75-cm^2^ flasks and used at 90% confluence. The day before the assay cells were detached from the flask with trypsin/EDTA (Sigma) and resuspended in 10 ml of Leibovitz’s L-15 media supplemented with 1% FBS, 100 U penicillin/streptomycin, and 15 mM glucose. The cells were plated in volume of 90 μl in black walled, clear bottomed 96-well microplates (Corning) and incubated overnight at 37°C in ambient CO_2_.

Membrane potential was measured using a FLIPR Membrane Potential Assay Kit (blue) from Molecular Devices, as described previously (Knapman et al., 2013). The dye was reconstituted with assay buffer [145 mM NaCl, 22 mM HEPES, 0.338 mM Na_2_HPO_4_, 4.17 mM NaHCO_3_, 0.441 mM KH_2_PO_4_, 0.407 mM MgSO_4_, 0.493 mM MgCl_2_, 1.26 mM CaCl_2_, 5.56 mM glucose (pH 7.4, osmolarity 315 ± 5)]. Prior to the assay, cells were loaded with 90 μl/well of the dye solution without removal of the L-15. Plates were then incubated at 37°C at ambient CO_2_ for 60 min. Fluorescence was measured using a FlexStation 3 (Molecular Devices) microplate reader with cells excited at a wavelength of 530 nm and emission measured at 565 nm. Baseline readings were taken every 2 s for at least 2 min, at which time either drug or vehicle was added in a volume of 20 μl. The background fluorescence of cells without dye or dye without cells was negligible. Changes in fluorescence were expressed as a percentage of baseline fluorescence after subtraction of the changes produced by vehicle (DMSO, 0.1% final concentration) addition.

### Biotelemetric Measurement of Core Body Temperature

The effect of CUMYL-4CN-BINACA on the body temperature of adult male C57BL/6J mice was assessed using radiobiotelemetric implants. Biotelemetry transmitters (TA-F10, Data Sciences International, St. Paul, MN) were implanted in four mice as previously described (Banister et al., 2019). Briefly, the mice were anesthetized (isoflurane, 3% induction, 1–2% maintenance) and a rostro-caudal incision was made alone the midline of the abdomen and the transmitter was placed in the peritoneal cavity according to the manufacturer’s protocol. The wound was sutured closed and testing commenced following 10 days recovery. All experiments and animal care procedures were approved by The University of Sydney Animal Ethics Committee in accordance with the *Australian Code of Practice for the Care and Use of Animals for Scientific Purposes*.

During testing, the mice were singly housed in a climate-controlled room (22 ± 1°C) on a 12 h light/dark cycle (lights on from 0600 to 1800). The mice were habituated to intraperitoneal injections of vehicle (7.8% polysorbate 80, 92.2% saline; injection volume 10 ml/kg) over multiple days at a set time (1100). The final habitation injection served as a drug-free baseline to which subsequent doses were compared. The mice were then administered CUMYL-4CN-BINACA at the same time of day in an ascending dose sequence (0.03, 0.1, 0.3, and 1 mg/kg), and body temperature data were recorded continuously using Dataquest A.R.T. software (Data Sciences International). The starting 0.03 mg/kg dose was selected based on the dose–response relationships of similar carboxamide-type SCRAs (Banister et al., 2019). Two drug-free washout days were given between each dose. For antagonist testing, an additional four mice were implanted with biotelemetry receivers. The mice were pretreated with either vehicle (the same mixture as specified above) or 3 or 30 mg/kg SR141716 (rimonabant; CB_1_ receptor antagonist) 30 min prior to a dose of 0.3 mg/kg CUMYL-4CN-BINACA (pretreatment order counter-balanced).

### Measurement of Seizure Behavior and Locomotor Activity

During biotelemetric testing, the experimenters noted some abnormal seizure-like behavior (Straub tail, myoclonic jerks) that occurred immediately following injections of 0.3 and 1 mg/kg CUMYL-4CN-BINACA. To quantify this behavior, drug naïve mice (not implanted with radiotelemetry probes) were injected i.p. with 0.3 mg/kg CUMYL-4CN-BINACA and placed in an observation chamber where video recordings were captured. Additional mice were administered vehicle solution (as specified in the section Biotelemetric Measurement of Core Body Temperature) and recorded under the same conditions. To assess the involvement of CB_1_ and CB_2_ receptors, further cohorts of mice were pre-treated with either 3 or 30 mg/kg SR141716, or 3 mg/kg AM-630, 30 min prior to vehicle or 0.3 mg/kg CUMYL-4CN-BINACA. An experimenter blinded to the experimental conditions scored the seizures using a modified Racine scale (Racine, 1972). Mice were scored with the following stages: 1) Straub tail; 2) rear leg twitches, 3) myoclonic jerks, 4) loss of posture, and 5) generalized tonic-clonic seizures. Seizure activities were counted and weighted by the above numbers (i.e., Straub tail counts multiplied by one, rear leg twitches by two, and so on) to yield a total Racine score, where higher numbers indicate greater seizure severity. Locomotor activity during the first 30 min postdosing was also quantified from the video recordings using automated tracking software (TopScan, CleverSys, Reston, VA).

### Statistical Analysis

For radioligand binding, corrected counts were exported and analyzed utilizing Prism (GraphPad Software Inc., San Diego, CA). The “one-site fit *K*_i_” model was used, specifying radioligand *K*_d_ as 1 nM for both CB_1_ and CB_2_. No other constraints were applied. Functional activity data were analyzed with Prism, using four-parameter nonlinear regression to fit concentration-response curves. A full CP 55,940 concentration response curve was completed every day, and a maximally effective concentration of CP 55,940 (1 µM) was included in every column of every plate to facilitate comparisons between experiments.

For *in vivo* biotelemetry, body temperature data were collated into 15-min bins using Dataquest A.R.T. software. Using Prism, each dose was compared to vehicle (or vehicle + CUMYL-4CN-BINACA for antagonist experiments) *via* an area under the curve (AUC) analysis over 3 h post-injection. Areas were analyzed using a one-way repeated-measures ANOVA with post hoc Dunnett’s contrasts.

For the behavioral observation experiments, mean Racine scores and locomotor activity were analyzed *via* a one-way ANOVAs, with Dunnett’s contrasts comparing the vehicle + 0.3 mg/kg CUMYL-4CN-BINACA condition to the remaining conditions.

## Results

**Figure 2:**
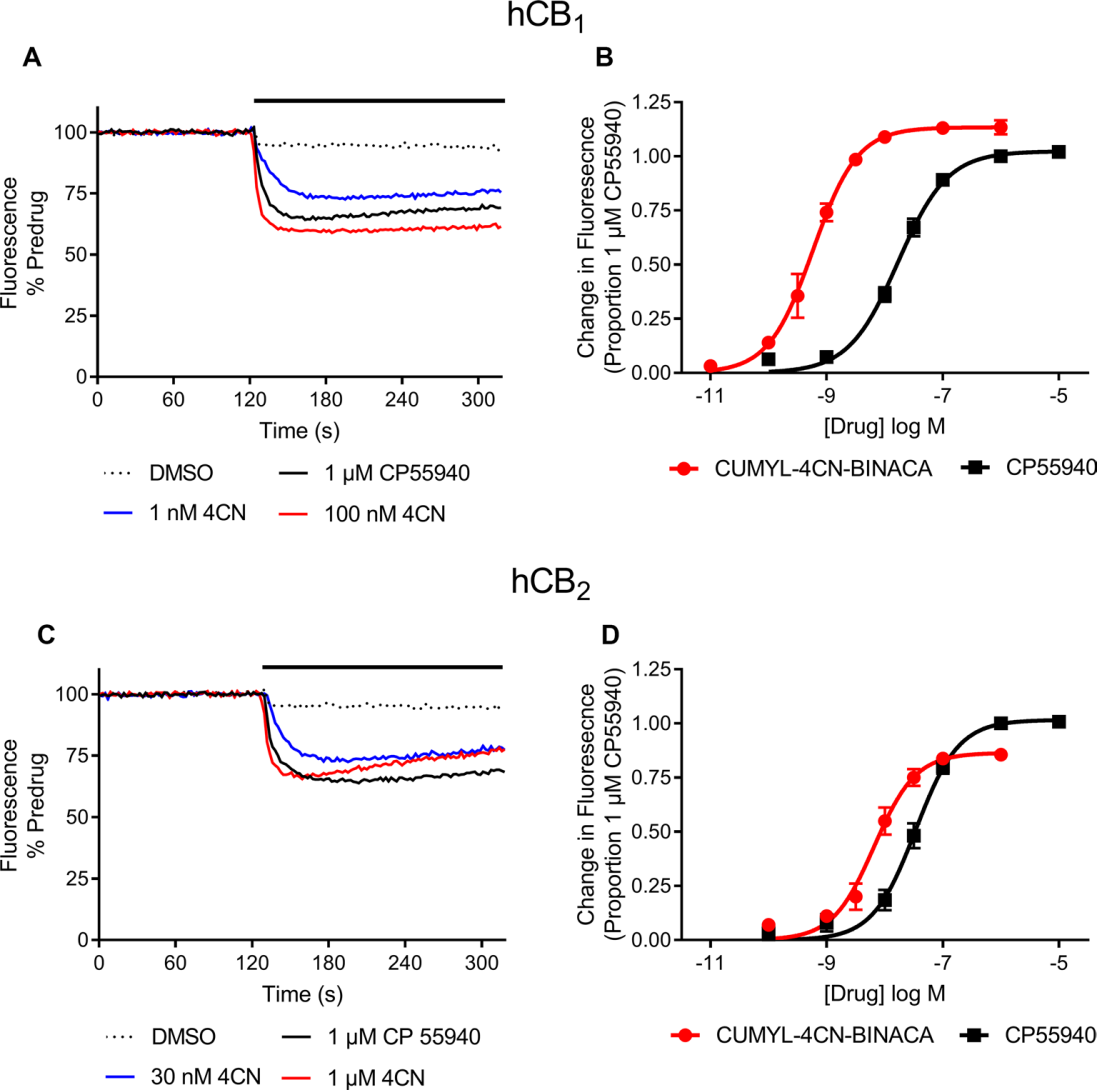
Representative traces of individual experiments illustrating the change in fluorescence in **(A)** AtT20-CB1 and **(C)** AtT20-CB2 cells following addition of CUMYL-4CN-BINACA (4CN) and CP 55,940, and concentration response curves for activation of **(B)** CB_1_ and **(D)** CB_2_ receptors by CUMYL-4CN-BINACA (4CN) and CP 55,940 in AtT20 cells. For representative traces, a drop in fluorescence represents an efflux of membrane potential sensitive dye from cells as they hyperpolarize. The data are presented as fluorescence normalized to the average of the fluorescence 30 s immediately prior to drug addiction. The effects of vehicle (DMSO) addition are illustrated, and have not been subtracted from the drug traces. For concentration response curves, the drug response is expressed as a proportion of that to CP 55,940, 1 µM. Data points represent the mean ± SEM of at least six independent experiments, and were fit to a four parameter logistic equation.

*In vitro*, CUMYL-4CN-BINACA functioned as a potent CB_1_ and CB_2_ receptor agonist (EC_50_ 0.58 nM and 6.12 nM at human CB_1_ and CB_2_ receptors, respectively) with nanomolar binding affinity (*K*_i_ 2.6 nM and 14.7 nM at human CB_1_ and CB_2_ receptors, respectively; **Table 1**). CUMYL-4CN-BINACA had a higher maximum response at CB_1_ receptors than CP 55,940, suggesting it may have higher intrinsic activity (**Figure 2**). It possesses similar binding affinity and functional activity to the closely related 5-fluoropentyl analogue 5F-CUMYL-PINACA, and both are more potent than CP 55,940 in the functional assay of GIRK activation (**Table 1**).

**Table 1:** Binding affinities and functional activities of CUMYL-4CN-BINACA and 5-fluoropentyl analogue 5F-CUMYL-PINACA at human CB_1_ and CB_2_ receptors.

Compound	hCB_1_			hCB_2_		
	p*K*_i_ ± SEM (*K*_i_, nM)	pEC_50_ ± SEM (EC_50_, nM)	Max ± SEM (% CP 55,940)	p*K*_i_ ± SEM (*K*_i_, nM)	pEC_50_ ± SEM (EC_50_, nM)	Max ± SEM (% CP 55,940)
CUMYL-4CN-BINACA (**1**)	8.58 ± 0.13 (2.6)	9.24 ± 0.04 (0.58)	113 ± 2	7.80 ± 0.02 (14.7)	8.21 ± 0.06 (6.12)	83 ± 2
5F-CUMYL-PINACA (**2**)	8.53 ± 0.04 (2.95)^a^	9.37 ± 0.06 (0.43)^b^	110 ± 3^b^	9.12 ± 0.12 (0.76)^a^	7.95 ± 0.09 (11.2)^b^	87 ± 3^b^

^a^Data extracted from Banister et al. (2019). ^b^Data extracted from Longworth et al. (2017).

**Figure 3:**
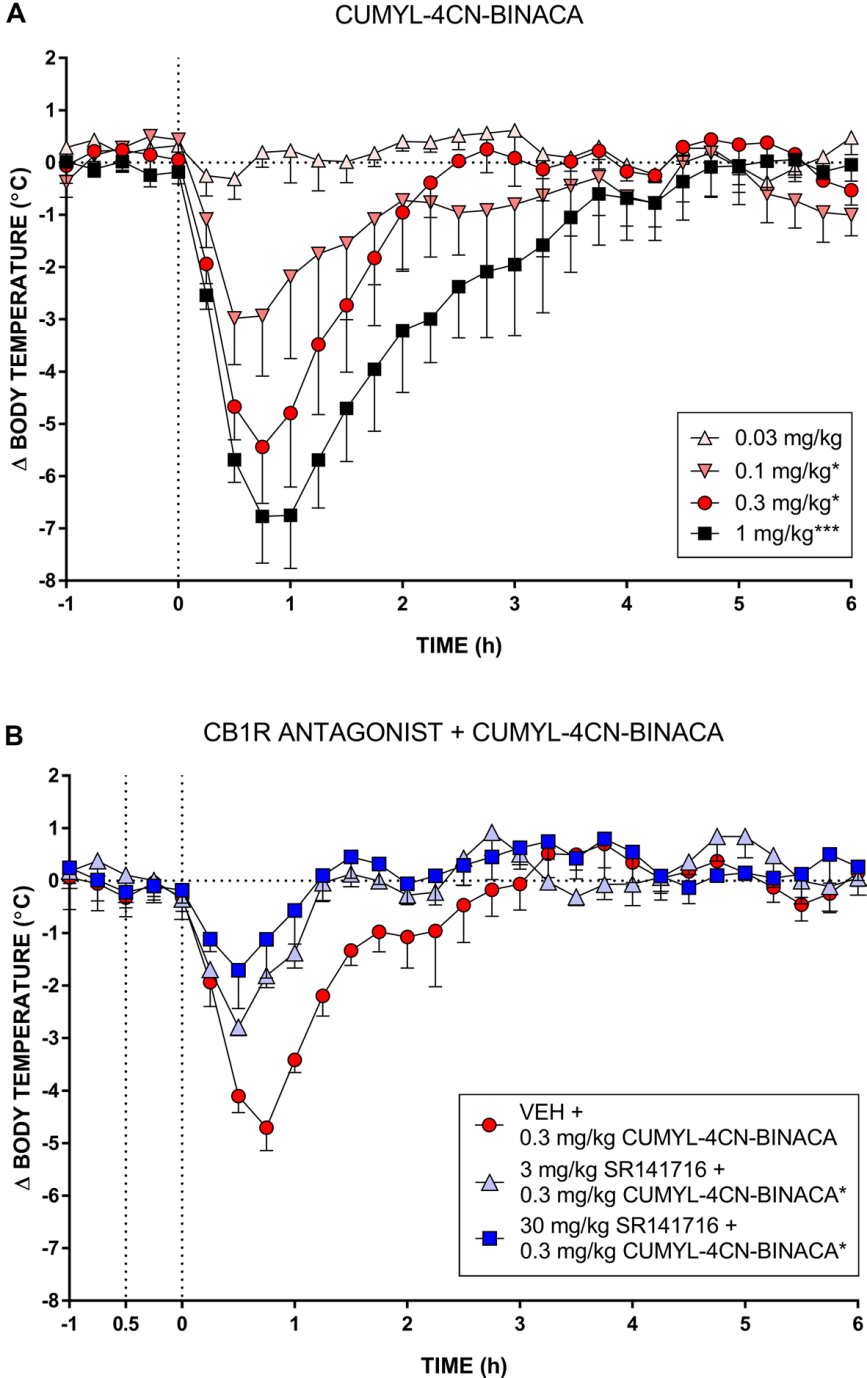
(**A)** Effect of CUMYL-4CN-BINACA on mouse core body temperature, where the dashed vertical line denotes time of intraperitoneal injection. *p < .05, ***p < .001 compared to vehicle, AUC over 3 h post CUMYL-4CN-BINACA injection. **(B)** Effect of 0.3 mg/kg CUMYL-4CN-BINACA on mouse core body temperature following pretreatment (30 min prior, first vertical dashed line) with vehicle or 30 mg/kg SR141716 (rimonabant; CB_1_ receptor antagonist). *p < .05 compared to vehicle + CUMYL-4CN-BINACA, AUC over 3 h post CUMYL-4CN-BINACA injection. For both **(A)** and **(B)**, each point represents the mean change in body temperature from vehicle baseline (± SEM) for four animals.

In mice, CUMYL-4CN-BINACA evoked a substantial hypothermic effect at doses of 0.1 mg/kg and higher (**Figure 3**). This effect increased in a dose-dependent manner. At the highest dose tested (1 mg/kg), we observed a peak reduction in body temperature of approximately 7°C. Higher doses were not administered given the strength of this effect. AUC analysis showed an overall statistically significant effect of dose (*F*_(4,12)_ = 14.18, *p* < 0.001), and the 0.1, 0.3, and 1 mg/kg doses differed significantly from vehicle (*p* < .05, *p* < .05, and *p* < .001, respectively, *post hoc* Dunnett’s contrasts). Pretreatment with 3 or 30 mg/kg of CB_1_ receptor antagonist SR141716 partially blocked the hypothermic effects of 0.3 mg/kg CUMYL-4CN-BINACA. AUC analysis showed an overall effect of antagonist pretreatment (*F*_(2,6)_ = 7.92, *p* < .05), and 3 and 30 mg/kg SR141716 pretreatment significantly reduced the hypothermic effect (*p* < .05 for both SR141716 doses, by *post hoc* Dunnett’s contrasts) compared to vehicle pretreatment.

**Figure 4:**
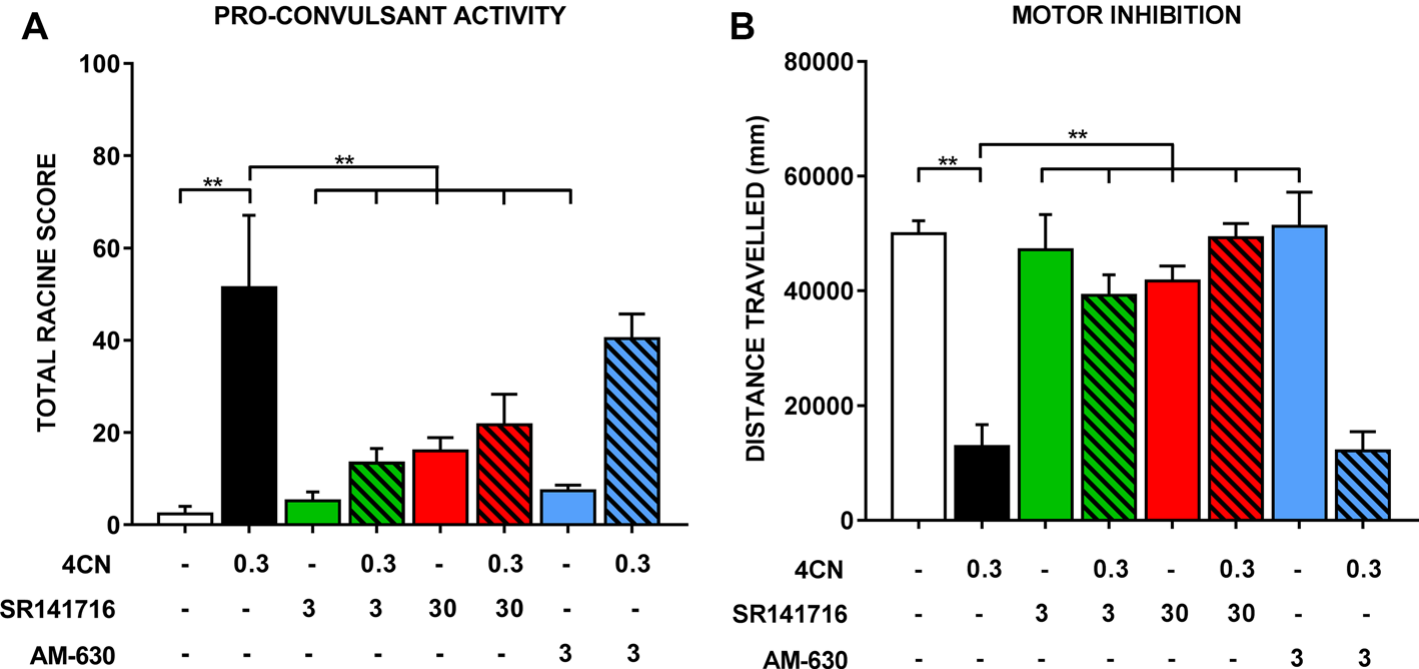
Seizure activity following an intraperitoneal injection of 0.3 mg/kg CUMYL-4CN-BINACA (4CN), pre-treated with vehicle, SR141716 (3 or 30 mg/kg), or AM-630 (3 mg/kg). Data are presented as **(A)** total Racine scores computed from weighted sums of each seizure behavior, which are presented individually in **Table 2**. Generalized tonic-clonic seizures were quantified but not observed under any condition. Locomotor data are presented in panel **(B)**. Bars represent mean ± SEM, with n = 5–6 per group; **p < .01, one-way ANOVA with Dunnett’s contrasts, comparing each condition to the vehicle + 0.3 mg/kg CUMYL-4CN-BINACA condition.

During radiobiotelemetric testing, myoclonic jerks and Straub tail were observed, in addition to a “gasping” reaction (not quantified), at doses of 0.3 and 1 mg/kg. These behaviors were observed shortly after injection (within 2–3 min) and lasted up to 1 h post-injection. Quantification of seizure activity showed an overall significant effect of drug treatment (F_(7,35)_ = 8.82, *p* < .0001), such that 0.3 mg/kg CUMYL-4CN-BINACA showed a clear and statistically significant (*post hoc* Dunnett’s test *p* < 0.0001) drug-effect compared to vehicle-treated mice (**Figure 4**). Straub tail and myoclonic jerks were consistently observed at this dose, in addition to a smaller number of instances of loss of posture; however, no generalized tonic-clonic seizures were observed (**Table 2**). This dose also produced substantial motor inhibition; the overall effect of drug treatment was statistically significant (F_(7,35)_ = 16.25, *p* < .0001), and locomotor activity was reduced following 0.3 mg/kg CUMYL-4CN-BINACA treatment compared to vehicle (*p* < .0001; **Figure 4**).

**Table 2:** Pro-convulsant behavioral distribution following CUMYL-4CN-BINACA (4CN) dosing with SR141716 or AM-630 pretreatment.

Pretreatment (mg/kg)	Drug treatment (mg/kg)	Straub tail	Rear leg twitch	Myoclonic jerk	Loss of posture
VEH	VEH	0.0 (0.0)*	1.2 (0.8)	0.0 (0.0)*	0.0 (0.0)
**VEH**	**4CN (3)**	**12.6 (3.1)**	**0.0 (0.0)**	**12.2 (5.3)**	**1.6 (0.5)**
SR141716 (3)	VEH	0.0 (0.0)*	2.6 (1.0)	0.0 (0.0)*	0.0 (0.0)
SR141716 (3)	4CN (3)	2.2 (0.5)*	4.2 (0.5)	1.0 (1.0)*	0.0 (0.0)
SR141716 (30)	VEH	0.0 (0.0)*	8.0 (1.4)*	0.0 (0.0)*	0.0 (0.0)
SR141716 (30)	4CN (3)	0.3 (0.2)*	10.7 (3.3)*	0.0 (0.0)*	0.0 (0.0)
AM-630 (3)	VEH	1.2 (0.3)*	2.9 (0.6)	0.0 (0.0)*	0.0 (0.0)
AM-630 (3)	4CN (3)	7.5 (1.8)	0.0 (0.0)	9.1 (1.9)	1.3 (0.8)

*p < .05 compared to vehicle + 3 mg/kg CUMYL-4CN-BINACA condition.

Pretreatment with SR141716 reduced total Racine scores (a higher score indicates greater pro-convulsant activity). Following pretreatment with 3 or 30 mg/kg SR141716, total seizure score was significantly reduced compared to vehicle pretreatment (*p* < .01 for both doses; **Figure 4**). Specifically, instances of Straub tail and myoclonic jerks were reduced (**Table 2**), suggesting a CB_1_ receptor mediated mechanism of action. However, rear leg twitches were observed with 3 mg/kg SR141716 treatment. When mice were pre-treated with a higher 30 mg/kg dose of SR141716 seizure activity resulting from 0.3 mg/kg CUMYL-4CN-BINACA (Straub tail and myoclonic jerks) was prevented. However, 30 mg/kg SR141716 treatment further increased the number of rear leg twitches and resulted in a greater total Racine score than that of the lower 3 mg/kg dose. 3 mg/kg AM-630 pretreatment did not significantly reduce total Racine scores (*p* > .05). Both 3 and 30 mg/kg SR141716 pretreatment blocked the effect of 0.3 mg/kg CUMYL-4CN-BINACA on locomotor activity (*p* < .01 and *p* < .0001, respectively), whereas 3 mg/kg AM-630 pretreatment had no effect (*p* > .05).

## Discussion

CUMYL-4CN-BINACA is a potent and efficacious CB_1_ receptor agonist *in vitro*, with an efficacy at least as great as CP 55,940, as is the case for several cumylamine-derived SCRAs (Longworth et al., 2017). It also possesses appreciable affinity and efficacy at CB_2_ receptors. Whereas nitrile SCRA AM-2232 has greater CB_1_ receptor binding affinity than its 5-fluoropentyl analogue AM-2201 (Makriyannis and Deng, 2001), CUMYL-4CN-BINACA has comparable CB_1_ receptor binding affinity and functional activity to its 5-fluoropentyl analogue 5F-CUMYL-PINACA. The nitrile modification, therefore, appears to only increase CB_1_ receptor binding affinity in select circumstances, presumably involving interaction with other structural elements of SCRA compounds.

*In vivo*, CUMYL-4CN-BINACA evoked strong hypothermic effects at doses as low as 0.1 mg/kg, positioning it as one of the most potent SCRAs tested in rodents to date. Related cumylamine-derived SCRAs produce similar hypothermic effects in rodents; CUMYL-PICA and 5F-CUMYL-PICA elicit hypothermia in rats at 1 mg/kg and above (Kevin et al., 2017; Longworth et al., 2017), and CUMYL-P7AICA produces a brief but intense hypothermic effect in mice at doses of 0.3 mg/kg and above (Banister et al., 2019). A partial blockade of the hypothermic effect of CUMYL-4CN-BINACA by SR141716 suggests involvement of CB_1_ receptors. This blockade was not total, possibly due to the high potency of CUMYL-4CN-BINACA, although we cannot rule out involvement of hitherto unidentified non-CB_1_ “off-targets.” It will be interesting to examine the effects of CUMYL-4CN-BINACA and related compounds in CB_1_ or CB_2_ knockout mice.

Pro-convulsant effects of several SCRAs have been observed in both human users and in animal models. For example, PB-22, AB-CHMINACA, and MDMB-CHMICA have been associated with generalized seizures in humans (Gugelmann et al., 2014; Hermanns-Clausen et al., 2018). In mice, 10 mg/kg 5F-AB-PINACA produces convulsions, which can be reduced by 10 mg/kg SR141716 pretreatment (Wilson et al., 2019). AM-2201 (2 mg/kg, i.p). induced epileptiform behaviors in mice in addition to abnormal spike wave discharges, which were suppressed by pretreatment with the CB_1_ receptor antagonist AM-251 (Funada and Takebayashi-Ohsawa, 2018). Similarly, JWH-018 produced electrographic seizures in mice at 1.5 mg/kg and above (Malyshevskaya et al., 2017), and convulsions at 6 mg/kg (Vigolo et al., 2015). The same study found that 10 mg/kg delta-9-tetrahydrocannabinol (Δ^9^-THC), the principal psychoactive component of cannabis, elicits electrographic seizures. In the present study, CUMYL-4CN-BINACA was pro-convulsant at 0.3 mg/kg i.p. To our knowledge, this is the most potent pro-convulsant SCRA reported to date.

Interestingly, this emerging evidence contrasts the historical use of cannabis and its phytocannabinoid constituents for the treatment of epilepsy and seizures (Rosenberg et al., 2015; Perucca, 2017). In particular, Δ^9^-THC has anti-convulsant properties in numerous rodent models (Karler et al., 1974; Dwivedi and Harbison, 1975; Sofia et al., 1976; Sofia and Barry, 1977) at very high doses (50 mg/kg and above). Additionally, the efficacious, non-selective CB_1_ agonist WIN-55,212-2 demonstrated anti-convulsant activity in mice *via* a CB_1_ receptor-mediated mechanism (Wallace et al., 2001). The pro- and anti-convulsant effects resulting from CB_1_ receptor activation may be dependent on dose (e.g., low versus high dose Δ^9^-THC), but have also been attributed to CB_1_ receptor modulation of both excitatory and inhibitory neurotransmission (Vilela et al., 2017). For example, activation of CB_1_ in inhibitory GABAergic interneurons could produce net excitation. Nevertheless, the precise mechanism(s) behind SCRA pro-convulsant effects remains to be elucidated.

SR141716 (3 mg/kg) did not completely block the pro-convulsant effects of CUMYL-4CN-BINACA, and although we were able to eliminate instances of Straub tail and myoclonic jerks with the higher 30 mg/kg dose of SR141716, we did observe seizure activity (rear leg twitches) with SR141716 treatment alone. The total Racine score of 30 mg/kg SR141716 was greater than the lower 3 mg/kg antagonist dose, suggesting a dose-dependent pro-convulsant effect of SR141716, with a distinct behavioral phenotype to CUMYL-4CN-BINACA. SR141716 and similar CB_1_ antagonists (e.g., AM-251) can function as inverse agonists at high doses, and like CB_1_ receptor agonists, possess both pro-convulsant and anti-convulsant properties in rodents depending on dose and seizure model. For example, SR141716 is pro-convulsant in the kainic acid-induced seizure model in mice (Marsicano et al., 2003), and in pilocarpine-induced seizure models in rats (Wallace et al., 2003), but anti-convulsant in rat hippocampal slices treated with glutamate receptor agonists (Karr et al., 2010).

In humans, a case report describes a partial seizure induced by SR141716 in a person with a history of epilepsy (Braakman et al., 2009), suggesting pro-convulsant activity following CB_1_ receptor antagonism in people with preexisting seizure vulnerability. Generally, SR141716 and related CB_1_ receptor antagonists/inverse agonists appear to be pro-convulsant in several seizure models. However, since these compounds are typically examined in combination with other drugs or as parts of larger seizure models, their independent convulsant properties are poorly characterized. In the present study, the pro-convulsant effects of SR141716 were subtle except at a very high dose (30 mg/kg) and in combination with CUMYL-4CN-BINACA. It is difficult to demonstrate a complete blockade of the pro-convulsant effects produced by a potent CB_1_ agonist like CUMYL-4CN-BINACA without also observing antagonist/inverse agonist mediated effects using currently available selective CB_1_ receptor antagonists. Nevertheless, SR141716 prevented the specific convulsant behaviors (Straub tail, myoclonic jerks, and loss of posture) observed following administration of CUMYL-4CN-BINACA alone, providing evidence that CB_1_ receptors are involved in the pro-convulsant effects of CUMYL-4CN-BINACA *in vivo*. CB_2_ receptor antagonist AM-630 failed to prevent the pro-convulsant or motor inhibitory effects of CUMYL-4CN-BINACA, suggesting that the CB_2_ receptor does not mediate these effects.

Human CUMYL-4CN-BINACA poisonings have been associated with altered mental status, anxiety, nausea and vomiting, seizures or shaking, and even death (European Monitoring Centre for Drugs and Drug Addiction, 2017; Horth, 2018). In these poisoning cases, doses have not been reported, however, given the low dose required to reliably produce seizure activity in mice compared to other SCRAs, and the observation of seizures or shaking in humans, we suggest that the CB_1_ receptor mediated pro-convulsant properties of CUMYL-4CN-BINACA identified here may contribute to the human toxidrome. This may be particularly evident in cases where product composition is heterogeneous (i.e., a “bad batch”), or where dosing has been estimated using non-quantitative techniques (e.g., measuring powders by eye), causing users to inadvertently administer a high dose.

### Conclusions

*In vitro*, CUMYL-4CN-BINACA is a highly potent and efficacious CB_1_ and CB_2_ receptor agonist with nanomolar binding affinity. Similarly, in mice, CUMYL-4CN-BINACA is a potent CB_1_ receptor agonist that produces dose-dependent hypothermia at doses of 0.1 mg/kg and higher, in addition to pro-convulsant behavior at doses of 0.3 mg/kg and higher. Pro-convulsant effects occurred at a substantially lower dose than those reported for other CB_1_ receptor agonists, which could contribute to the deleterious effects associated with human use of this compound.

## Ethics Statement

This study was carried out in accordance with the recommendations of The University of Sydney Animal Ethics Committee in accordance with the Australian Code of Practice for the Care and Use of Animals for Scientific Purposes. The protocol was approved by the University of Sydney Animal Ethics Committee.

## Author Contributions

RK, IM, MG, MC, and SB designed the study. RK and LA carried out the rodent pharmacology. RB and JM carried out the cellular pharmacology. RK and SB wrote the first manuscript draft. LA, RB, JM, MG, and MC wrote sections of the manuscript. All authors contributed to manuscript revision and approved the submitted version.

## Conflict of Interest Statement

The authors declare that the research was conducted in the absence of any commercial or financial relationships that could be construed as a potential conflict of interest.

## Funding

This work was supported by a National Health and Medical Research Council (NHMRC) Project Grant (1107088) to IM and MC, and by the Maurice and Phyllis Paykel Trust to MG.
